# Current Status of HbA1c Biosensors

**DOI:** 10.3390/s17081798

**Published:** 2017-08-04

**Authors:** Hua Lin, Jun Yi

**Affiliations:** School of Environmental and Biological Engineering, Nanjing University of Science and Technology, Nanjing 210094, China; linhua@njust.edu.cn

**Keywords:** glycated hemoglobin (HbA1c), biosensor, boronic acid, antibody, fructosyl valine

## Abstract

Glycated hemoglobin (HbA1c) is formed via non-enzymatic glycosylation reactions at the α–amino group of βVal1 residues in the tetrameric Hb, and it can reflect the ambient glycemic level over the past two to three months. A variety of HbA1c detection methods, including chromatography, immunoassay, enzymatic measurement, electrochemical sensor and capillary electrophoresis have been developed and used in research laboratories and in clinics as well. In this review, we summarize the current status of HbA1c biosensors based on the recognition of the sugar moiety on the protein and also their applications in the whole blood sample measurements.

## 1. Introduction

With the worldwide improvement in the living conditions of humans in general, accompanied by problematic changes in diet and life-style habits such as reduced exercise, diabetes mellitus (DM) has become a major epidemic disease. According to the latest report by the International Federation of Clinical Chemistry and Laboratory Medicine (IFCC), there were 415 million people with diabetes around the world, of which 47% of the patients were unaware of their state of illness [1].

Glycated hemoglobin (HbA1c) is a typical glycosylated protein in the body, and its abundance reflects the average blood glucose level over two to three months, corresponding to the 100- to 120-day lifespan of erythrocytes. The HbA1c level can not only be used by diabetes patients to monitor their long-term glucose management in a way that is not affected by fluctuations of the blood-glucose level, but also can be used by doctors to assess potential risks of diabetes complications of patients. In 2010 and 2011 the American Diabetes Association (ADA) and World Health Organization (WHO) have recommended a diagnostic cut-off point of ≥6.5% HbA1c as one of three diagnostic criteria for diabetes, respectively [2,3].

The glycosylation of hemoglobin occurs via a sequential two-step non-enzymatic reaction. Firstly, the neutral amino groups from N-terminal residues or from the side chain of Lys residues in hemoglobin (Hb) interact with the aldehyde or ketone groups in sugar molecules to form reversible Schiff base intermediates. Then the intermediate undergoes an irreversible intermolecular Amadori rearrangement to generate a more stable ketoamine structure [4]. The probabilities of glycosylation are dependent on the local pKa of the amino groups as well as the charge and steric effects by neighboring residues [5]. In general, the pKa values of α–amino groups of N-terminal residues, especially the one of βVal1 (close to 7), are lower than those of ε–amino groups of Lys residues in Hb. In addition, a positively charged cavity around the βVal1 moiety has a strong affinity to attract sugar molecules. Due to the high concentration of glucose in the blood stream, the most abundant form of the glycosylated hemoglobins (GHbs) is the glucose adduct named HbA1c.

The HbA1c level is defined as the ratio of HbA1c to the total Hb concentration, and the physiological range of HbA1c in the whole-blood samples is 3–13 mg/mL in terms of concentration or 5% to 20% in terms of percentage of total Hb. The ADA-recommended diagnostic criteria of HbA1c for diabetes and prediabetes are shown in Table 1 [6]. For pregnant diabetic individuals, it is strongly recommended that they have a stringent control on their HbA1c to minimize risks such as congenital malformations, overweight infants, and complications of pregnancy [7]. Accurate and precise methods to detect HbA1c are thus required for better diagnosis and management control of DM.

In the past several decades, a variety of HbA1c detection methods has been developed, such as immunoassay [8], ion-exchange chromatography [9], boronate affinity chromatography [10], electrophoresis [11,12], and a colorimetric method [13]. Those methods can be generalized into three basic chemical principles based on charge differences, structural differences and chemical reactivity. Most of these methods are subject to at least one or more types of interferences from Hb variants including various forms of Hb (HbC, HbS, HbE, HbD, and HbF) and/or the modification of Hb (cabamylated Hb, acetylated Hb, labile HbA1c) [14]. The global standardization of HbA1c was conducted by the IFCC and evaluated by different designated comparison methods developed by the National Glycohemoglobin Standardization Program (NGSP) and others, resulting in a laboratory-based gold reference method of HbA1c [15]. Although the IFCC reference method can provide precision and accuracy for HbA1c measurement [16], the needs for sophisticated equipment and the professional personnel to operate the system limit its accessibility to only large medical organizations or research institutions [17]. Therefore, it is valuable to develop methods that are easy to operate and cost-effective, but robust enough for clinical use. HbA1c biosensors have great potential for the design of analytical devices with appropriate sensitivity, low cost, simplicity, and possibility for miniaturization. Recently, several very good review articles have summarized a wide range of methods being used for the determination of glycosylated proteins including HbA1c [18,19,20,21,22,23]. In this review, we summarize recent progress in the development of HbA1c biosensors from several aspects, including the prototypical methodology for HbA1c measurement, applications in whole-blood sample analysis, and the development of point-of-care (POC) technology in this research field, including non-invasive biosensors for diabetes.

## 2. HbA1c Biosensors

The recognition components of electrochemical HbA1c biosensors can be divided into three categories: boronic acid derivatives, antibodies, and enzymes. This review will report on all three, below, and what follows here is a summary. Current progress on the most popular boronic acid-based electrochemical biosensors is summarized first, according to the type of boronic acid derivative employed, electrode-surface modification, and the detection method. Antibody-based immunobiosensors are mainly focused on immobilization of components, either HbA1c or the HbA1c-specific antibody, and the subsequent detection of HbA1c. The working principle of the third type known as enzymatic reaction-based biosensors will be briefly introduced third.

### 2.1. Affinity Biosensors Based on Boronic Acid Derivatives

**Figure 1:**
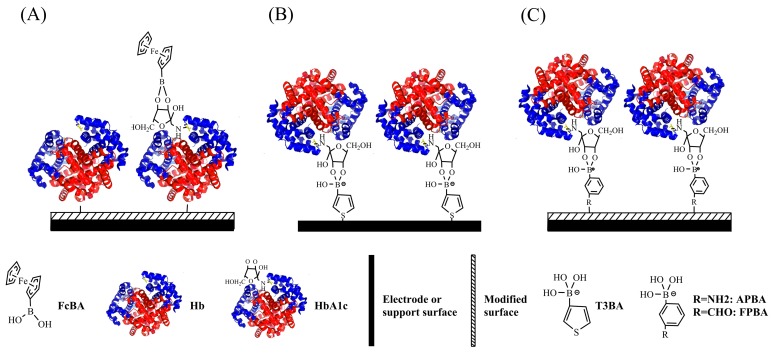
Schematic illustration of construction of boronic acid-based HbA1c sensors by using (**A**) FcBA; (**B**) T3BA; and (**C**) APBA or FPBA for HbA1c recognition.

Boronic acid derivatives have a high affinity for the *cis*-diol group of the sugar moiety in glycosylated proteins, which is the fundamental principle behind several affinity-based methods for detection of glycosylated proteins including HbA1c. Here we summarize the methods, which will be elaborated below. One way is to bind Hbs (both HbA and HbA1c) by haptoglobin (Hp) on an electrode surface first, and then ferrocene-boronic acid (FcBA) is added to the system. The FcBA, which can be probed by electrochemical signals, will interact with only HbA1c, not Hb, (Figure 1A). The second method is to immobilize boronic acid derivatives on the electrode, resulting in a selective binding of HbA1c, by which an appropriate electrical signal can be detected (Figure 1B,C).

#### 2.1.1. Biosensors Based on Ferrocene-Boronic Acid (FcBA)

FcBA plays a dual role in the determination of HbA1c. First, the boronic acid portion of the molecule interacts with HbA1c. Second, the ferrocene portion is a redox-active label, therefore, it can be utilized for both target recognition and signal production. By using a pyrolytic graphite electrode (PGE), that is coated with zirconium dioxide (ZrO_2_) nanoparticles (NP)/didodecyldimethyl-ammonium bromide (DDAB) interface, Liu et al first immobilized Hbs on the electrode, and then incubated the protein-bound electrode in FcBA solution. The binding of the FcBA to HbA1c generates a clear redox peak monitored by cyclic voltammetry, and the peak current of the bound FcBA increases linearly with HbA1c levels in the range of 6.8~14% [24]. However, the adsorption of Hbs onto ZrO_2_/DDAB interface is time-consuming and the statistical deviation of the method is relatively high by a comparison with the results measured by the reference method. Further improvement is required in terms of the response time and precision for the clinical application.

Halámek and coworkers tested various Hbs-adsorbent materials including surfactants, a strong cation propidium modifier, an organophosphorous compound and Hp as well. The deoxycholic acid (DOCA)-modified surface showed the best overall performance based on three parameters: loading capacity of the modifier, reproducibility of the piezoelectric sensor and the direct Hb reduction. The total Hb content deposited on the surface was monitored using a quartz crystal microbalance (QCM), while the HbA1c concentration was obtained by subsequent voltammetric detection of FcBA coupled on the sensor surface, in which the signal is proportional to the HbA1c concentration [25]. Due to the heat-denaturation of the Hb pretreatment and a pH-dependent high standard deviation value of the method, currently, this is still a HbA1c-assay concept.

Recently, quite a few of boronic acid-based biosensors have been developed for the capture and determination of HbA1c including 3-aminophenylboronic acid (APBA), thiophene-3-boronic acid (T3BA), and formylphenylboronic acid (FPBA), as summarized in Table 2. In order to attach boronic acids to the electrode surface, one must modify them with cross-linking agents. For example, the electrodes can be modified with the cysteamine self-assembled monolayer (SAM), followed by glutaraldehyde and then APBA [26], to create the final boronate affinity layer (Figure 1C). There are other functions of cross-linking agents such as the ERGO-PQQ, ESM-glutaraldehyde, carboxy-EG6-undecanethiol and pTTBA-Au NPs used for the same purpose.

#### 2.1.2. Biosensors Based on Thiophene-3-Boronic Acid (T3BA)

Electrochemical impedance spectroscopy (EIS) has been widely used to characterize sequential chemical modifications on an electrode surface by measuring changes of conductivity or dielectric constant, and can be adopted for the design of label-free biosensors. The instrument puts a small oscillating voltage across the membrane over a range of frequencies. The resulting current and its phase shift are the data that provide information about the interface. For example, T3BA-SAM is bound to the gold electrode surface via the Au-S covalent bonds (Figure 1B). The formation of T3BA-SAM followed by HbA1c on the Au electrodes are confirmed and characterized by QCM, atomic force microscopy, and EIS methods [27]. The concentration of HbA1c on the T3BA-modified biosensors is measured by monitoring the changes of charge-transfer resistance, which are owed to the blocking of the electrode surface by the immobilized HbA1c, with hexacyanoferrate (HCF) as the redox indicator [27,28]. Some sensors are developed to utilize the capacitive behaviors of the electrode interface to measure the surface-bound HbA1c without a need of redox indicator [29]. However, the dynamic detection range of this method is yet to match the physiological range of HbA1c from 3 to 13 mg/mL.

#### 2.1.3. Biosensors Based on 3-Aminophenyl Boronic Acid (APBA)

Siva and coworkers reported the synthesis of a APBA-graphene oxide (GO) conjugate via the amide linkage confirmed by IR spectroscopy [30], and its application in the determination of glycosylated hemoglobin (GHb) by EIS. It appears that the high conductivity and wide surface area of GO enhance the performance of these APBA-GO modified biosensors.

Eggshell membranes (ESMs), a natural porous fiber, are used as a low-cost protein-immobilization platform. By using 3-APBA-modified ESMs biosensor, the HbA1c can be measured in a clinically relevant range (2.3~14%) with a detection limit of 0.19% [31]. The EIS biosensors exhibit good reproducibility, precision and selectivity. Although further optimization shortens the time of HbA1c measurement [32], the need for sophisticated instrumentation severely limits the EIS-based biosensor from being used for clinical application. In contrast, the features of simplicity and rapid measurement increase the possibility of the amperometric method for the design of portable devices.

Since the boronate-based biosensors lack selectivity between glycated Hb and sugar molecules, one can solve this by use of the heme iron as catalyst as well as by carrying out an adequate pretreatment of the sample to remove plasma interferences. Kim and coworkers have fabricated a disposable biosensor using APBA chemically bonded to a poly(terthiophenebenzoic acid) (pTTBA)/AuNPs-modified screen-printed electrode [33] (Figure 1C). The detection of HbA1c is by measurement of the response current of catalytic reduction H_2_O_2_ by the heme of the protein on the interface. The sensor has been used successfully for the analysis of simple filtration-prepared finger-prick blood samples, exhibiting a linear response to HbA1c levels of 0.1~1.5% in amperometric mode and 0.5~6% in impedometric mode, respectively. An indirect competitive assay for HbA1c determination was developed in which HbA1c competes with alizarin red S (ARS) for binding to phenylboronic acid (PBA) to form diol-boronic complexes in solution [34]. The potential shift of ARS is correlated with the competitive binding of HbA1c to PBA, and is proportional to the concentration of HbA1c. In addition, the total Hb concentration is measured using HCF as the redox indicator to give the result of % HbA1c in line with that measured by a reference method.

Although the above methods can provide direct electrochemical detection of HbA1c, they require addition of an exogenous redox indicator, such as a redox mediator or H_2_O_2_, due to the relative electrochemical inactivity of HbA1c itself. A label-free and enzyme-free HbA1c sensor was prepared by modifying the electrode surface with poly(3-aminophenylboronic acid) (PAPBA) NPs [35]. The peak current measured by differential pulse voltammogram (DPV) decreases with increasing HbA1c concentrations from 1.0 to 156 μM, owing to the fact that the HbA1c bonded with the PAPBA interface blocks the ion-flux-channel of the PAPBA NPs. The sensor also showed high selectivity toward HbA1c over glucose, serum albumin, ascorbic acid, uric acid, dopamine and Hb. A label-free voltammetric HbA1c sensor was fabricated by a composite multilayer of APBA-pyrroloquinoline quinone (PQQ) coupled with electrodeposition of reduced graphene oxide (ERGO) on the glassy carbon electrode (GCE) [36]. The APBA/PQQ/ERGO/GCE not only displays a high sensitivity for HbA1c determination with the linear range is 9.4~65.8 μg/mL, but also can be applied for whole blood samples.

Besides the conventional electrochemical detection methods, Chen and coworkers also employed a home-built surface plasmon resonance (SPR) biosensor to study interactions between the APBA monolayer and HbA1c as functions of pHs and salt concentrations [37]. The surface of an Au-coated SPR probe was modified with 4,4-dithiodibutyric acid (DTBA)-APBA monolayers in order to bind HbA1c. Under the optimized condition, the SPR signal shows a linear response to HbA1c in the range of 0.43–3.49 μg/mL and with the detection limit of 0.01 μg/mL.

#### 2.1.4. Biosensors Based on Formylphenylboronic Acid (FPBA)

More recently, to amplify the electrochemical signal, Song and coworkers developed a competitive binding assay, also named as an enzymatic backfilling assay, in which HbA1c is immobilized on a FPBA-modified interface first, then a known amount of glucose oxidase (GOx) is backfilled on the surface. Due to the reverse concentration correlation between HbA1c and GOx, the electrochemical signal of the GOx decreases with the increasing HbA1c level [38,39]. In their early work, poly(amidoamine) G4 dendrimer was used as the interface material to capture periodate-activated GOx, and the current of GOx decreases with increasing HbA1c level from 2.5% to 15% with a spiking method. In 2012, the cystamine-FPBA conjugate was used to functionalize a gold electrode surface in order to bind HbA1c and GOx competitively via cis-diol interaction. This method is applied for the HbA1c detection from whole blood samples. The pretreatment of Hbs via a Zn-induced precipitation with a follow-up washing-out step was carried out to remove interference of other glycoproteins or carbohydrates in whole blood. The HbA1c-GOx competitive binding assay on a cystamine-FPBA/Au electrode could measure HbA1c level in the range of 4.5~15% which covers the clinical reference range of diabetes without a need for detection labels.

### 2.2. Antibody-Modified Biosensors

**Figure 2:**
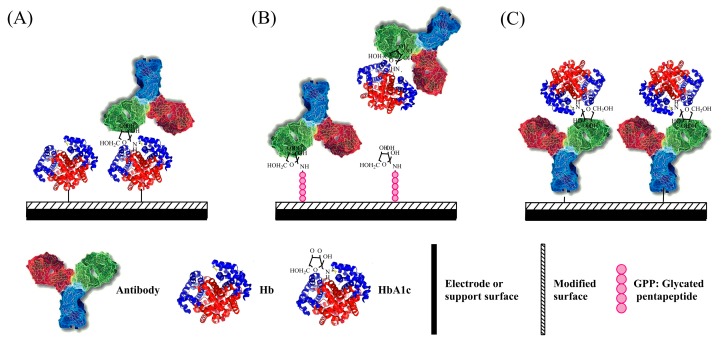
Schematic illustration of construction of HbA1c immunosensor by using (**A**) DOCA or Hp; (**B**) GPP; and (**C**) 3-MPA, PPy-Au NPs, and Mixed SAMs for HbA1c recognition.

Immunobiosensors based on the different immobilization components can be divided into three categories. One is similar to the Fc-modified electrode, where Hb and HbA1c are immobilized on the surface of an electrode without differentiation (as Figure 2A). The second type is to attach HbA1c analog on the electrode surface (as Figure 2B), which competes with HbA1c for the sites of HbA1c-specific antibody. The third type is a fixed antibody as the recognition and capturing element for HbA1c only (as Figure 2C). Comparison of determination features of HbA1c immunosensors based on the immobilization method is summarized in Table 3.

#### 2.2.1. Immobilization of Hbs on the Electrode Surface

Based on the design of DOCA-modified interface for Hbs immobilization [25], Halámek utilized anti-HbA1c immunoglobin G (IgG) to selectively bind to HbA1c and used FcBA which binds to multiple sugar moieties on IgG (Figure 2A) to amplify the electrochemical signal of FcBA [40]. The anodic peak height of the square wave voltammograms of FcBA is proportional to the bound IgG-FcBA which is correlated to the HbA1c present on the surface. The sensitivity of this electrochemical immunosensor is three times than that of the FcBA-based HbA1c sensors without the antibody.

Stöllner and coworkers developed a sandwich-type HbA1c sensor consisting of the first layer of a site-directed Hp-modified cellulose membrane for Hbs enrichment, after capturing the Hbs, and sequential addition of an anti-HbA1c antibody followed by the introduction of anti-IgG(sheep)-GOx for the specific detection of bound HbA1c [41]. Both the optical signal generated by the HbA1c-sandwich ELISA assay and the electrochemical responses generated from the Hp/anti-HbA1c-GOx/Clark-type electrode provide a linear correlation in the clinically relevant range of 5~20% HbA1c of total Hb. However, the current status of the HbA1c-sandwich immunoassay is still as a proof-of-concept design owing to the defects that include a single usage per membrane, long detection time needed, and a large dilution factor of blood sample required (up to 15,000-fold dilution) to minimize the non-specific binding of Hbs [41].

#### 2.2.2. Immobilization of a Glycated Pentapeptide as HbA1c Analogon

Similar to the ARS/PBS competitive inhibition assay mentioned in the boronic affinity section, an immunoenzymometric assay for HbA1c detection has been developed based on the competitive binding of the HbA1c analyte and the surface-bound glycated pentapeptide (GPP) epitope to a known amount of anti-HbA1c antibody [42]. The enzymatic activity of glucose oxidase-antibody conjugate measured by absorbance spectroscopy is proportional to the amount of surface-bound anti-HbA1c antibody for GPP, but in an inverse relationship to HbA1c concentration. The synthetic GPP analogon shares the same amino acid sequence of the first five residues of the N-terminal βHb. Compared to one time usage of the protein-modified cellulose membrane, the GPP-modified surface exhibits very good reproducibility and can be repetitively regenerated more than 20 times without loss of the binding affinity.

Liu and coworkers constructed a series of electrochemical immunosensors based on the competitive inhibition assay in which HbA1c competes with surface-bound GPP for binding to anti-HbA1c antibody. There are several key features of the electrode fabrication for those biosensors: (i) the GCE surface is coated with a mixed layer of oligo(phenylethynylene) molecular wire (MW) and oligo(ethylene glycerol) (OEG) [43] or with AuNP-diazonium salt modified interface [44,45]; (ii) in the amperometric immunosensor, the redox probe of 1,10-di(aminomethyl) ferrocene (FDMA) is covalently bound to the modified interface followed by the immobilization of GPP; (iii) Ru(NH_3_)_6_^3+^/Ru(NH_3_)_6_^2+^ redox couple is used in the EIS immunosensor based on AuNP-diazonium salt modified interface; (iv) the OEG molecules work as the insulator to prevent nonspecific protein binding on the electrode surface; (v) in the amperometric mode, the relative current increases with the increase of HbA1c concentration; (vi) in the impedenmetric mode, the change of electron transfer resistance shows an inverse linear correlation with the increase of HbA1c concentration in the sample. The dynamic range of HbA1c detection of those three types of electrochemical immunosensor covers the physiological range of HbA1c level from 4.5~15%.

#### 2.2.3. Immobilization of HbA1c Antibody

The immobilization of the anti-HbA1c antibody on the electrode surface is the most direct and specific way to bind HbA1c (Figure 2C). By covalently immobilizing HbA1c-specific antibody on the surface of a gold working electrode treated with the SAM of 3-Mercaptopropionic acid (MPA), the binding of HbA1c was monitored by differential pulse voltammetry (DPV) using ferricyanide/ferrocyanide as the redox mediator [46]. The single-use disposable three-electrode immunosensor shows a linear range of 7.5–20 µg/mL of HbA1c in 0.1 M phosphate buffer solution and 0.1–0.25 mg/mL of HbA1c in undiluted human serum. Hence, this biosensor has a great potential for application to in vitro measurement of HbA1c for diabetic management.

Xia and co-workers constructed a series of ion-sensitive field effect transistor (ISFET)-based HbA1c immunosensors composed of a FET-based sensor chip and a disposable extended-gate electrode chip by using complementary metal-oxide-semiconductor transistor (CMOS) and micro-electronic mechanical system (MEMS) techniques. Much effort has put on the method development of HbA1c/Hb antibody immobilization on the gold working electrode, including polypyrrole (PPy)-Au NPs composite film [47], mixed self-assembled monolayers (SAMs), seed mediated growth of nanogold film [48], and mixed SAMs wrapped with a nanogold array [49,50,51,52]. The PPy-Au NPs composite gold electrode fabricated by electro-polymerization technique exhibits a linear response over 4~18 μg/mL of HbA1c [47]. Although the response time is fast (less than 1 min), the stability and signal reproducibility of the sensor need further improvement.

The mixed SAMs wrapped with nano-gold array interface for antibody immobilization are prepared as follows: (i) form two-layer thiol-containing SAMs (16- and 3-mercaptohexadecanoic acid) on the gold nanospheres; (ii) modify the gold electrode with SAM of mercaptoethylamine; (iii) bioconjugate the mixed SAMs/Au nano-spheres with modified-Au electrode in the presence of NHS and EDC; (iv) and covalently bind the HbA1c or Hb antibodies on the mixed SAMs/Au electrode via the amide linkage in the presence of NHS and EDC.

The mixed SAMs/Au nanospheres array-modified electrode provides several valuable features. For example, the two-layer structure of SAMs on Au nano-spheres reduces the steric hindrance for biomolecule binding. Given the uniformly distribution of nano-spheres on the electrode surface with an increased large surface area-to-volume ratio, the sensitivity and signal reproducibility of this micro immunosensor are improved. In addition, by immobilizing HbA1c and HbA antibody separately, the FET-based immunosensors can simultaneously measure HbA1c and HbA concentrations without a separation procedure which has great advantages for the development of HbA1c POC device.

A sandwich immunoassay configuration is constructed by using specific monoclonal HbA_1c_ antibody to capture the antigen followed with the addition of high fluorescent CdTe quantum dots (QDs)-labeled polyclonal secondary antibodies to measure HbA1c by both optical and electrochemical means [53]. The QD-based electrochemical assay showed a linear increase in current with increasing levels of HbA1c (4–16%) and a high correlation coefficient of 96% compared to that of the standardized HPLC method. Furthermore, this method requires a very small sample volume and is applicable for rapid, reproducible, and cost-effective analysis of HbA1c in clinical samples.

### 2.3. Indirect HbA1c Biosensors Based on the Determination of Fructosyl Valine

HbA1c can be potentially digested to form fructosyl valine (FV) moiety, which can be measured quantitatively via an enzymatic reaction by fructosyl amino acid oxidase (FAO). There are three categories of FV-based biosensors of which applications are summarized in Table 4.

**Figure 3:**
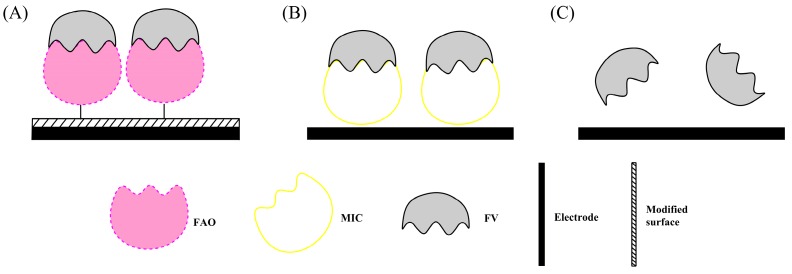
Schematic illustration of the construction of indirect HbA1c biosensors based on the recognition of fructosyl valine on the (**A**) PVA-SbQ or NPs modified electrode surface; (**B**) by MIC method; (**C**) by Non-enzymatic method.

The first type of indirect HbA1c sensors measures the electro-oxidation of FV by FAO which is immobilized on the electrode surface prior to the enzymatic reaction (Figure 3A). The second category involves construction of a molecular-imprinted polymer (MIP) as the artificial enzyme to oxidize the FV (Figure 3B). The third type is to direct measure the oxidation of FV using electrochemical sensors (Figure 3C).

#### 2.3.1. Fructosyl Amino Acid Oxidase (FAO)-Modified Biosensors

For the FAO-based biosensors, the enzyme can be either confined in the film made of poly(vinyl alcohol) cross-linked photochemically with stilbazole (PVA-SbQ) [54], or immobilized on the surface of nanoparticulate materials, such as iridium (Ir) NPs [55], Fe_3_O_4_ NPs [56], ZnO NPs [57], and Au NPs [58]. The NPs, PPy, and graphene nanosheet are promising materials to be used for the fabrication of the FAO-modified biosensors.

The FAO enzyme can react with both FV and ε-fructosyl lysine (ε-FL) substrates. This latter reaction would give a false positive, so it is not an ideal element for HbA1c enzyme-based biosensors. Sakaguchi and coworkers used a novel protein, fructosyl amino acid binding protein, to detect FV [66]. Its high sensitivity and specificity for FV over ε-FL makes the new sensing system ideally suited for the measurement of HbA1c. In addition, the protein engineering of FAOx was recently reviewed [67]. The FAO enzyme is still very expensive, and to obtain the FV will inevitably increase the cost and inconvenience for HbA1c assays.

#### 2.3.2. Molecular Imprinting Catalyst (MIC)-Modified Biosensors

Sode and coworkers constructed a MIC-based sensor using the molecular imprinting technology to create an artificial enzyme [60]. The MIC has a low selectivity to FV, so it is important to improve it to increase its selectivity. In addition to optimizing experimental conditions, Sode also used allylamine to increase the selectivity for FV [61], but the selectivity is still too low for practical detection. Recently, Yamazaki modified the Au electrode with a synthetic soluble molecular imprinting polymers (MIPs) to FV recognition [62]. Katterle and coworkers optimized the experiment by aligning PBA residues in a suitable orientation in MIPs for the binding of FV, resulting in a higher selectivity to FV compared to fructose and pinacol imprinting [68]. The same research group constructed a thermometric MIP sensor which can detect FV concentration in the range of 0.25–5 mM [63].

#### 2.3.3. Non-Enzymatic Biosensors

The FV concentration can also be measured in a non-enzymatic system. Chien and Chou constructed FcBA modified GCP electrode for both FV molecule recognition and electrochemical signal transduction [65]. The amperometric signal was linear for detection of FV concentrations at the mM level with negligible interference from glucose. The same authors tried to use a relatively high potential of 1.0 V to directly oxidize FV by a glassy carbon-paste electrode [64]. However, interfering compounds such as ascorbic acid could be oxidized at that potential and could give false readings.

## 3. Analysis of Samples from Human Blood

A good sensor system with potential for clinical use must be able to assess HbA1c level with the sensitivity and reproducibility while provide excellent operational and reagent-storage stability. The sensor system should be able to distinguish HbA1c from non-glycated hemoglobin (HbA0) or other interferences and demonstrate excellent agreement with a standard analytical method. Table 5 summarizes some the applications of biosensors in HbA1c measurement from whole-blood samples.

Most of the examples described in the section above mainly focus on the method development for HbA1c determination. For practical application, HbA1c content rather than HbA1c concentration is used, which is the ratio of HbA_1c_ concentration to total Hb concentration. Units for HbA1c content are commonly reported either in mmol/mol (used by the IFCC) or in percentage format (used by the NSGP). In general, a pretreatment of blood samples is needed to remove plasma interference followed by lysis of the RBCs. Hb and HbA1c are then measured separately using those biosensor systems.

An UV-Vis spectroscopy method can be used to quantify Hb concentration before the hemolysate sample is applied to biosensors for HbA1c readout [36]. The HbA1c content can easily be calculated from these numbers. However, this kind of detection method is inconvenient in practice because the detection and separation are distinct processes.

Halámek and coworkers have developed FcBA-based biosensors for the detection of HbA1c [25,40]. Hbs are adsorbed to the surfactant-modified surface first, and then followed by using the mass-sensitive quartz crystal balance, and also using voltammetry to monitor total Hb and HbA1c, respectively. HbA1c binding to the APBA-modified electrodes was reversible, thereby providing a reusable sensing system. As such, Halámek applied the feature to develop a novel HbA1c biosensor based on flow injection [72]. They used a reticulated vitreous carbon (RVC) electrode modified with 3-APBA to separate and detect the HbA1c concentration, followed by the detection of Hb using the screen-printed electrode modified with a sol-gel film involving chitosan, and tetraenthoxyl silica entrapped into carbon nanotubes. This sensor showed a good relation between signal and Hbs concentrations.

Even though gold has been commonly used in the biosensor system, carbon or membrane-based materials have been adopted into for their biocompatibility and cost effective feature. Booyasit constructed an 3-APBA-modified ESMs electrode to detect HbA1c and a Hp-modified ESMs electrode to determine the Hb [32]. These sensors can simultaneously detect total Hb and HbA1c with excellent precision and also an acceptable reproducibility of fabrication. The chemical stability of the Hp-modified ESMs is good (98.84% over a shelf-life of 4 weeks), however, for the APBA-modified ESMs, the stability is not so good (92.35% over a one-week period). Thus one needs to improve the stability of the APBA-modified ESM for clinical application.

In addition to the electrochemical signal transduction pathway, optical detection methods are also used in the HbA1c biosensor systems. Ahn and coworkers fabricated a HbA1c-capturing interface made of carboxy-EG6-undecanethiol SAM coupled with 3-APBA on a gold thin-film substrate, and used an 11-amino-1-undecanethiol SAM modified gold substrate for Hb immobilization [73]. The chemical luminescence (CL) response in the Luminol/H_2_O_2_ system in which the four heme groups play a role as the catalyst is linearly proportional to the amount of Hbs. The luminol CL method not only provides a high sensitivity for HbA1c detection without the need for signal amplification, but also can be used for Hb detection on an amino-SAM based interface, which means this method can be applied on whole blood sample analyses. The linear dynamic range of HbA1c level is from 2.5% to 17% which covers the clinical concentration range with negligible interference from other carbohydrates.

One of the main advantages of the boronate affinity-based method is that the measurement does not interfere with Hb variants such as HbF, HbS, HbC, formayl-Hb etc., which makes it applicable to a large population. In addition, boronate-based reagents are relative stable and cost-effective compared to HbA1c antibodies. Due to the nature of the boron-cis-diol covalent bond, this method actually measures GHb (HbA1c and Hb glycated at other Lys sites) and also interferes with other endogenous sugar molecules in the sample.

Because of its high specificity and selectivity, the HbA1c antibody is the best candidate for capturing HbA1c. Xia and coworkers developed a micro immunosensor consisting of an ISFET integrated chip (see Section 2.2.3 above) and an electrode array based on the micro-electro-mechanical systems (MEMS) technology, modified with anti-Hb and anti-HbA1c antibodies [51]. The sensor can simultaneously detect the concentration of HbA1c and Hb to obtain HbA1c level. The responses of the immunosensor are linear over the concentration range of 166.7–570 ng/ml Hb and 50~170.5 ng/ml HbA1c which are 10^5^-fold lower than physiological Hb concentrations. To apply the method for whole blood analysis, the samples were lysed and diluted 150,000-fold prior to measurements. This micro immunosensor exhibited a low relative deviation of measured HbA1c level. By reducing cost, enhancing the shelf-life of the antibody, and invoking lab-on-a-chip (LOC) technology, electrochemical immunosensors will have a great potential to be translated into clinical use.

Moon and coworkers developed a disposable microfluidic amperometric dual-sensor for the detection of HbA1c and total Hb separately [74]. The concentration of total Hb was measured by the cathodic currents of total Hb catalyzed by a TBO/pTTBA@MWCNT-modified working electrode. On the other surface of the dual-sensor, aptamer was used for HbA1c immobilization. After removal of unbound Hb by washing with PBS, the cathodic current of HbA1c captured on the surface was determined by the aptamer/TBO/pTTBA@ MWCNT-modified working electrode in the fluidic channel.

Given the advantages of minimal sample volume, rapid analysis time and wide accessibility to diagnosis, the global POC diagnosis market is expected to reach US$36.93 billion by 2021 (http://www.marketsandmarkets.com/PressReleases/point-of-care-diagnostic.asp). More than a dozen commercial HbA1c POC devices are currently used in clinics, of which the principal detection method is either based on boronate affinity separation or immunoassay. Among most cited devices, In2it^TM^ (Bio-Rad, Hercules, CA, USA), Alere Afinion^TM^ (Alere Technologies AS, Oslo, Norway), Nycocard (Alere Technologies AS) and Clover A1c (Inforpia, Kyunggi, Korea) are built based on the affinity method, while DCA Vantage (Siemens Medical Solutions Diagnostics, Tarrytown, NY, USA) and A1CNow series (Bayer HealthCare, Sunnyvale, CA, USA) are based on the immunoassay method. In a very recent review, Hirst and coworker independently reviewed 1739 records published before June 2015 in several databases (Medline, Embase and Web of Science) and carried out a meta-analysis on sixty-one studies to compare the accuracy and precision of eleven HbA1c POC devices [75]. The analysis results show that the majority of devices (9 out of 11) have a negative mean bias compared to laboratory comparator methods, as well as a large variability in bias within devices. The implication of using HbA1c POC testing results on medical treatment decision-making and patient outcomes needs to be evaluated further.

## 4. Conclusions

With the number of diabetic patients increasing each year, it has caused a huge burden on public medical resources. A HbA1c POC device with good analytical performance can help patients with diabetes to monitor their long-term glycemic status. More importantly, the characteristics of immediate feedback can avoid delaying the diagnosis and treatment of diabetes. The boronate affinity-based method and the immunoassay method are the two methodologies currently used in commercially available HbA1c POC devices. Given the advantages of being relatively simple to operate, easy to miniaturize as well as its high sensitivity, the electrochemical HbA1c biosensors can be potentially translated for clinical use.

Other noninvasive technologies have emerged to supplement current blood glucose and HbA1c measurements for diabetes diagnosis and management. For example, Saraoglu developed a QCM sensor by an electronic breath-analysis system to detect the amount of acetone in exhaled breath for indirectly determining HbA1c levels and blood glucose for 30 patients’ samples [76]. The average accuracy rates for HbA1c level and blood glucose predictions are approximate 83% and 75%, respectively. With an expected global market of up to US $12.2 billion by the end of 2017, blood glucose monitoring occupies the top share in the POC market [77]. Raman scattering has also become a promising tool for noninvasive, continuous tracking of blood glucose level [78,79]. By exploiting an improved concentration independent calibration approach, Spegazzini and coworkers performed a longitudinal tracking of blood glucose which exhibited a 35% reduction in error compared to a conventional calibration method [79].

The integrations of affinity- and immunoassay-based HbA1c biosensors with emerging cell phone-based technology and LOC platforms, supplemented by noninvasive blood glucose monitoring provide a highly promising interface for the design of new generation of personalized HbA1c POC devices [77].

## Figures and Tables

**Table 1:** ADA criteria for the diagnosis of diabetes and prediabetes based on HbA1c.

Status	HbA1c (%) ^a^	HbA1c (mmol/mol) ^b^
Prediabetes	5.7~6.4	39~47
Diabetes	≥ 6.5	≥ 48

**^a^** suggested by the Diabetes Control and Complications Trial (DCCT); **^b^** suggested by the IFCC.

**Table 2:** Comparison of HbA1c biosensors based on boronic acid derivatives.

Type of Boronic Acid	Modified Element/Electrode/Label or Redox Indictor	Detection Method	Dynamic Range ^a^ (μg/mL or %)	Limit of Detection (μg/mL)	Reference
FcBA	ZrO_2_ NPs/PGE	Amperometric	6.8–14%	ND	[24]
	DOCA-4-aminothiophenol monolayer/Au	Voltammetric	0–20%	ND	[25]
T3BA	Au/HCF	Impedimetric	0.1–1	ND	[27]
	Au/IgG-FITC	Impedimetric	10–100	1	[28]
	Au/anti-HbA1c-IgG-FITC	Impedimetric	10–100	ND	[29]
APBA	glutaraldehyde-SAM Cys/IDAs/HCF	Impedimetric	0.10–8.36%	0.024%	[26]
	GO/GCE/HCF	Impedimetric	10.32–61.92	ND	[30]
	glutaraldehyde-ESMs/Screen printed Pt/HCF	Impedimetric	2.3–14%	0.19–0.21%	[31,32]
	pTTBA-Au NPs/SPE	Impedimetric/Amperometric	0.5–6.0%/0.1–1.5%	0.052%	[33]
	PBA-ARS/GCE/HCF	Potentiometric	5.0–50	ND	[34]
	PAPBA NPs-thin film/SPCE/PAPBA	Amperometric	63–10062 (0.975–156 μM)	ND	[35]
	PQQ-ERGO/GCE/PQQ	Voltammetric	9.4–65.8	1.25	[36]
	SAM DTBA/Au	SPR	0.43–3.49	0.1	[37]
FPBA	SAM cystamine/Au-Si/FcM & GOx	Voltammetric	4.5–15%	ND	[38]
	poly(amidoamine) G4 dendrimer/Au/FcM & GOx	Voltammetric	2.5–15%	ND	[39]

**^a^** μg/mL for HbA1c concentration only, % for the concentration ratio of HbA1c to HbA.

**Table 3:** Comparison of HbA1c immunosensors based on recognition methods.

Types	Modified Element/Electrode/Label or Redox Indictor	Detection Method	Dymamic Range (μg/mL)	Reference
Immobilized Hbs	DOCA/Au/FcBA	Voltammetric	ND	[40]
	Hp/cellulose membrane or microtiter/TMB & anti IgG-GOx	Amperometric	0–25% (7.8–39 nM)	[41]
Competitive inhibition	GPP/microtiter plate/TMB & anti IgG-GOx	Photometric	1.5–10 (1 nM)	[42]
	Oligo(phenylethynylene) MW-FDMA-GPP/GC/FDMA	Amperometric	4.5–15.1%	[43]
	Au NPs -FDMA-GPP/GCE/FDMA	Amperometric	4.6–15.1%	[44]
	Au NPs-GPP/GCE/Ru(NH_3_)_6_^3+/2+^	Impedimetric	0–23.3%	[45]
Immobilized anti-HbA1c Antibody	SAM 3-MPA/Au/HCF	Voltammetric	7.5–20	[46]
	Ppy-Au NPs /Au/PPy	Potentiometric	4–18	[47]
	SAM 1,6-hexanedithiol Au NPs/Au/HCF	Potentiometric	4–24	[48]
	mixed SAMs/Au/HCF	Potentiometric	ND	[49]
	mixed SAMs wrapped nano-spheres array/Au/HCF	Potentiometric	0.050–0.1705	[49,50,51,52]
	seed mediated growth nano-gold/Au/HCF	Potentiometric	0.00167–0.07214	[52]
	Protein A/laser ablated Au electrode/anti HbA1c-QDs	Optic-electrochemical	ND	[53]

**Table 4:** Comparison of FV sensors based on different oxidation methods.

Type	Modified Element/Electrode/Label or Redox Indictor	Detection Method	Dynamic Range (mM)	Limit of Detection (uM)	Applied Potient (V)	Reference
Immobilized FAO	PVA-SbQ/Pt	Amperometric	0.2–10	200	0.6	[54]
	Ir NPs/Carbon	Amperometric	0–0.5	ND	0.25	[55]
	Fe_3_O_4_ NPs/Au/HCF	Amperometric	0.1–2	100	ND	[56]
	ZnO NPs-Ppy/Au/HCF	Amperometric	0.1–3	100	ND	[57]
	Au NPs-GNs/FTO Glass Plate	Impedimetric	0.0003–2.0	0.2	0.2	[58]
MIC	PVI/CPE/m-PMS	Amperometric	0.02–0.7	20	0.1	[59]
	MIC/CPE/m-PMS	Amperometric	0.2–0.8	ND	ND	[60]
	MIC-Allylamine/ND/m-PMS	Amperometric	ND	ND	0.1	[61]
	MIC/Au/m-PMS	Amperometric	0.05–0.6	ND	ND	[62]
	MIP	Thermometric	0.25–5.0	ND	ND	[63]
Non enzymatic	None/GCPE/ITO glass	Amperometric	0–1.0	ND	1	[64]
	None/GCE/FcBA	Amperometric	0.1–4.0	500	0.1	[65]

**Table 5:** HbA1c biosensors for whole blood sample measurement.

Biosensors			Detection Method	Dynamic Range (μg/mL)		Ref.
HbA1c/HbA0	Electrode	Label/Redox Indictor	HbA1c/HbA0	HbA1c	HbA0	
ESMs-APBA/Hp-ESMs	SPCE	HCF	Impedimetric	2.3–14%	5000–200,000	[32]
APBA-PQQ-ERGO/none	GC	PQQ	Voltammetric/Photometric	9.4–65.8	92.5	[36]
DOCA-4-aminothiophenol monolayer/none	Au	FcBA or anti-HbA1c Ab	Voltammetric/Piezoelectric	0–20%	ND	[25,40]
mixed SAMs wrapped nano-spheres array-anti-HbA1c/anti-HbA0 Ab	FET	HCF	Potentiometric	0.05–0.1705	0.167–0.570	[51]
ABPA-mercaptoundecanoic acid/none	Au	ND	Piezoelectric/Photometric	3–11% 50–2000	10–90	[69]
m-APBA agarose beads/none	IDAs	HCF	Electrochemical	ND	ND	[70]
APBA-column/none	GC	Ferrocene/FC-Ab	Amperometric	4–12.6%	0–500	[71]
APBA/sol-gel film	RVC/SPE	ND	Amperometric	200–12,000	20,000–200,000	[72]
carboxy-EG6-undecanethiol-APBA/11-amino-1-undecanethiol	Au	Luminol/H_2_O_2_	Chemiluminescence	2.5–17% 2–80	50–200	[73]
MWCNT-pTTBA-TBO & aptamer/TBO	SPCE	TBO	Amperometric	0.387–484	6.45–645	[74]

## Abbreviations

ADA	American Diabetes Association
APBA	3-aminophenyl boronic acid
ARS	alizarin red S
Au	gold
CPE	carbon paste electrode
DCCT	Diabetes Control and Complications Trial
DDAB	didodecyl-dimethylammonium bromide
DM	diabetes mellitus
DOCA	deoxycholic acid
DTBA	4,4-dithiodibutyric acid
EIS	electrochemical impedance spectroscopy
ERGO	electrodeposition of reduced graphene oxide
ESMs	eggshell membranes
FAO	fructosyl amino acid oxidase
FcBA	ferrocene-boronic acid
FcM	ferrocenemethanol
FDMA	1,10-di(aminomethyl) ferrocene
FET	field effect transistor
FITC	fluorescein isothiocyanate
FPBA	formyl-phenylboronic acid
FTO	fluorine doped tin oxidase
FV	fructosyl valine
GCE	glassy carbon electrode
GCPE	glassy carbon paste electrode
GHbs	glycosylated hemoglobins
GNs	graphene nanosheets
GO	graphene oxide
GOx	glucose oxidases
GPP	glycated pentapeptide
Hb	hemoglobin
HbA0	non-glycated hemoglobin
HbA1c	glycated hemoglobin
HCF	hexacyanoferrate
Hp	haptoglobin
IDA	interdigitated electrode array
IFCC	International Federation of Clinical Chemistry and Laboratory Medicine
IgG	immunoglobin G
ISFET	ion-sensitive field-effect transistors
ITO	indium tin oxide
LOC	lab-on-a-chip
LOD	limit of detection
MEMS	micro-electro-mechanical systems
MIC	molecular imprinting catalyst
MIPs	molecular imprinting polymers
MPA	3-Mercaptopropionic acid
MWCNT	multi-wall carbon nanotube
NGSP	National Glycohemoglobin Standardization Program
NPs	nanoparticles
OEG	oligo(ethylene glycol)
PAPBA	poly(3-aminophenylboronic acid)
PGE	pyrolytic graphite electrode
POC	point-of-care
PPy	polypyrrole
PQQ	pyrroloquinoline quinone
pTTBA	poly (2,2′:5′,5″-terthiophene-3′-p-benzoic acid)
QDs	quantum dots
RVC	reticulated vitreous carbon
SAM	self-assembled monolayer
SPCE	screen-printed carbon electrode
SPE	screen-printed electrode
SWV	square wave voltammograms
T3BA	thiophene-3-boronic acid
TBO	toluidine blue O
TMB	3,3’,5,5’-tetramethylbenzidine
WHO	World Health Organization
ZnO	zinc oxide
ZrO_2_	zirconium dioxide
